# Effect of Compaction Degree on the Carbonation Properties of Steel Slag

**DOI:** 10.3390/ma18071629

**Published:** 2025-04-03

**Authors:** Zihan Yan, Wenxiao Fu, Longbin Zhao, Ziyan Gao, Sitong Chen, Qianruo Wang, Wei Long

**Affiliations:** 1 School of Materials and Chemical Engineering, Hubei University of Technology, Wuhan 430068, China; yanzh204117@163.com (Z.Y.); fuwxxx@163.com (W.F.); gaozyyyyyy@163.com (Z.G.); chenzayne@163.com (S.C.); wangqianruoooo@163.com (Q.W.); 2 Key Laboratory of Green Materials for Light Industry of Hubei Provincial, Wuhan 430068, China; 3 Hubei Engineering Laboratory of Automotive Lightweight Materials and Processing, Wuhan 430068, China

**Keywords:** compaction degree, carbonation, carbonation products, microstructure

## Abstract

Carbonation technology offers a novel approach to enhance steel slag performance, where the compaction degree plays a pivotal role in optimizing the carbonation process. This study reveals that as the compaction degree increases, the peak temperature in the carbonation environment gradually decreases, and the intensity of the carbonation reaction weakens. Post-carbonating, the compressive strength initially increases before declining, peaking at a compaction degree of 60%. At this optimal compaction degree, the material achieves a compressive strength of 124.4 MPa and a CO_2_ uptake of 14.5%. The analysis of pore size distribution and carbonation products reveals that steel slag compacts with lower compaction degrees exhibit larger internal pores, leading to dispersed and isolated carbonation products, which restrict performance improvement. Conversely, excessively high compaction degrees cause the premature blockage of gas diffusion pathways by calcium carbonate particles, which impede the carbonation process and degrade the mechanical performance. The moderate compaction of steel slag effectively prevents the early blockage of gas channels, and significantly facilitates the accumulation and bonding of carbonation products, thereby achieving the superior performance.

## 1. Introduction

With the rapid advancement of global industrialization, resource consumption and environmental issues have become increasingly prominent. Effectively utilizing industrial solid waste has become a key focus for both academia and industry [1,2,3]. Steel slag, a by-product of steel manufacturing, is endowed with considerable potential for resource recovery, owing to its distinctive physical, chemical, and mineralogical characteristics, such as enhanced mechanical properties, including superior compressive and shear strength, as well as high wear resistance, while containing reactive components such as C_2_S, C_3_S C_4_AF, and C_3_A [4,5,6]. A study indicates that utilizing steel slag as a substitute for natural aggregates can reduce concrete production costs by 15–20% compared to conventional crushed stone aggregates, while simultaneously decreasing environmental costs associated with mining [5]. As steel production continuous to escalate, the generation of steel slag has correspondingly increased. The inadequate management of this material not only occupies substantial land resources but also poses severe environmental risks [7,8]. Nevertheless, steel slag is abundant in reactive components such as silicates, aluminates, and calcium oxide, which confer excellent mechanical properties and latent cementitious capabilities. These attributes render it highly suitable for diverse applications such as construction materials [9], soil enhancement [10], and water purification [11].

In recent years, the efficient utilization of steel slag resources has become a critical pathway for advancing waste minimization, resource recovery, and environmentally safe disposal. This approach holds significant importance for achieving green development and fostering a circular economy. Carbonation technology (A technical system that involves controlled chemical reactions between carbon dioxide (CO_2_) and alkaline substances to form stable carbonate minerals), as an innovative method that integrates industrial solid waste with carbon dioxide, has demonstrated great potential in enhancing the utilization of steel slag resources [12,13,14]. The alkaline components in steel slag react with carbon dioxide to form stable minerals such as calcium carbonate. This reaction not only enhances the solidification and value-added properties of steel slag but also facilitates the sequestration of carbon dioxide [15,16]. Consequently, this process significantly improves the mechanical properties and environmental durability of steel slag, enabling its widespread application in the production of concrete aggregates [15,17], subgrade materials [18], and functional fillers [19,20]. However, due to the complex composition of steel slag and the multi-phase reaction mechanisms involved in carbonation, its carbonation performance is influenced by a variety of factors, including reaction conditions, material structure, and external environments [21,22].

A comprehensive investigation into the microscopic mechanisms and optimization strategies of steel slag carbonation is of great scientific significance and practical value for promoting the efficient application of carbonation technology in steel slag resource utilization [23,24,25,26,27]. In recent years, numerous studies have examined the influence of forming pressure on the carbonation performance of steel slag. For example, Yang et al. found that the compressive strength of carbonated steel slag initially increased and then decreased with rising forming pressure, a trend attributed to microstructural transformation induced by the combined effects of carbonation and compaction [28]. Similarly, Xu et al. successfully fabricated steel slag carbonated boards with mechanical properties comparable to natural limestone by optimizing key parameters such as the water-to-solid ratio, porosity, CO_2_ pressure, and sodium carbonate content during the preparation process [22]. P. Nielsen explored the factors affecting the development of the compressive strength of the steel slag carbonation-solidified body, including carbonation conditions, material ratios, curing time, and other key parameters [29]. Despite these advancements, there remains a notable research gap regarding the influence of the compaction degree on the carbonation process and the underlying mechanisms governing the performance evolution of steel slag.

This study aims to elucidate the mechanism by which the compaction degree influences the carbonation process and performance enhancement of steel slag, thereby providing theoretical foundations and technical guidance for optimizing its resource utilization. As a critical parameter governing the pore structure of steel slag, the compaction degree directly impacts the diffusion and reaction efficiency of carbon dioxide, and significantly determines the distribution of carbonation products and the resulting mechanical property changes. A deep understanding of the compaction mechanism not only sheds light on the intrinsic patterns of pore filling and structural densification during carbonation, but also provides a scientific basis for optimizing industrial process parameters, such as carbonation duration, CO_2_ concentration and pressure, and carbonation environment temperature. For instance, the optimization of the carbonation duration ensures a sufficient CO_2_ diffusion and complete reaction; controlling the CO_2_ concentration and pressure enhances the uniformity and efficiency of the carbonation reaction; and the precise regulation of the carbonation environment temperature directly affects the rate and stability of carbonate formation. This research not only promotes the efficient application of steel slag in construction materials and environmental remediation but also contributes significantly to achieve carbon reduction and carbon neutrality goals.

## 2. Experimental

### 2.1. Materials

**Figure 1:**
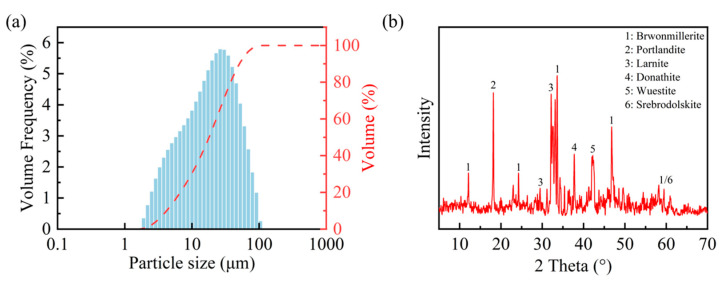
Particle size distribution (**a**) and mineral composition (**b**) of steel slag powder.

The steel slag powder utilized in this study was obtained from an alkaline oxygen furnace supplied by a firm based in Wuhan, Hubei. Before conducting the experiment, the steel slag powder was dried at 60 °C for 24 h to eliminate residual moisture. The density of the steel slag powder was measured to be 3.551 g/cm^3^ via the pycnometer method (the pycnometer method is a density measurement technique grounded in Archimedes’ principle, where the sample density is calculated through the precise volumetric displacement measurement of a reference liquid within a calibrated vessel), and its specific surface area was measured at 349 m^2^/kg using a Micromeritics ASAP 2460 automatic surface area analyzer (Micromeritics, Norcross, GA, USA). Figure 1a illustrates the particle size distribution of the steel slag powder ranging from 1.905 µm to 104.729 µm. Table 1 and Figure 1b present the chemical composition and major mineralogical constituents of the steel slag powder, revealing three predominant crystalline phases including Larnite (Ca_2_SiO_4_), Srebrodolskite (Ca_2_Fe_2_O), and Portlandite (Ca(OH)_2_).

### 2.2. Experimental Processes

In this study, the compaction degree of steel slag compacts was systematically controlled by regulating the mass of the steel slag powder while maintaining a constant specimen height of 20 mm. The compaction degree, calculated according to Equation (1) [22], was selected as the primary experimental variable to evaluate its influence on the carbonation kinetics. Five distinct compaction degrees (50%, 55%, 60%, 65%, and 70%) were examined, with the corresponding mass requirements for each condition detailed in Table 2. For the preparation of the steel slag compacts, the steel slag powder was initially homogeneously mixed with deionized water at a controlled water-to-solid ratio of 1:10 (*w*/*w*). Subsequently, the required quantities of the moistened mixture for each compaction degree were precisely weighed and transferred into a cylindrical mold (20 mm diameter × 20 mm height). The compaction process was conducted under constant pressure for a duration of 90 s, followed by careful demolding to obtain cylindrical specimens.

*Degree of compaction* = (*m*/*ρ*)/(*πD*^2^/4 × *h*) × 100% (1)

where *m* is the mass of steel slag in the compact before carbonation, g; *ρ* is the density of steel slag, g/cm^3^; *D* is the diameter of the compact, cm; and *h* is the height of the compact, cm.

### 2.3. Carbonation

**Figure 2:**
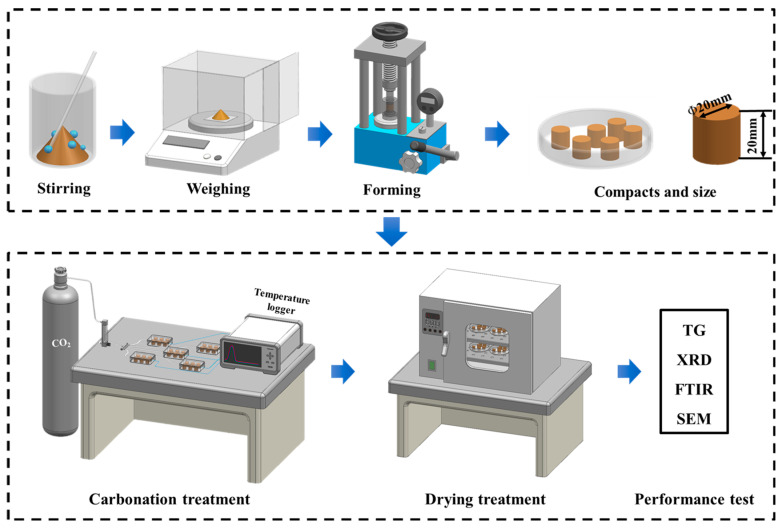
Flow chart of preparation and carbonation of steel slag compact.

After demolding, the steel slag compacts were immediately subjected to a carbonation curing treatment. Specimens with different compaction degrees were individually placed in gas-tight reaction chambers maintained at a CO_2_ concentration of 99.9% with a constant pressure at 0.1 MPa. The carbonation system was equipped with a gas flow meter to the ensure rigorous control of the reaction environment. To systematically investigate the temporal evolution of carbonation, four distinct curing durations (1, 3, 6, and 9 h) were implemented. After curing, the steel slag compacts were dried at 60 ± 2 °C for 6 h. Subsequently, a comprehensive characterization was performed to evaluate the mechanical properties, phase composition, and microstructural evolution of the carbonated compacts. The complete experimental procedure, encompassing specimen preparation and carbonation treatment, is schematically illustrated in Figure 2.

### 2.4. Test Methods

#### 2.4.1. Carbonation Ambient Temperature

The carbonation reaction chamber is connected to a data logger (AT4610, Changzhou Applent, Changzhou, China) (Figure 2), and the carbonation environment temperature is monitored and recorded in real-time using a thermocouple and the data logger. The sampling frequency is set to 1 s, and the temperature is continuously recorded for 9 h of carbonation.

#### 2.4.2. Compressive Strength and CO_2_ Uptake

The compressive strength is tested using the microcomputer control electronic universal testing machine (WDW-100M, Jinan Zhonglu Chang, Jinan, China), with a loading rate of 2 mm/min. The reported compressive strength values represent the mean of triplicate measurements obtained from three identical compacts within each experimental group. The CO_2_ uptake is calculated using Equation (2) [13].

(2) $$CO_{2}uptake=\frac{{mass}_{post-carbonated}+{mass}_{collectedwater}-{mass}_{pre-carbonated}}{{mass}_{solid}}\times 100\%$$

#### 2.4.3. Microscopic Analysis

The microstructural characterization of steel slag compacts was conducted through a comprehensive suite of analytical techniques. The pore structure evolution of steel slag compacts during the carbonation was quantitatively assessed using an automatic mercury porosimeter (Micromeritics AutoPore V 9600 (Micromeritics, Norcross, GA, USA)) with a measurable pore diameter range of 5 nm to 350 µm. Prior to analysis, specimens were subjected to vacuum drying at 60 °C for 24 h to ensure complete moisture removal.

The carbonation products were quantitatively analyzed via a thermogravimetric differential scanning calorimeter (NETZSCH TG209F3, NETZSCH Analyzing & Testing, Selb, Germany). The thermal analysis was performed under a nitrogen atmosphere with a heating rate of 10 °C/min over a temperature range of 30–800° C.

A phase composition analysis was conducted using an X-ray diffraction (XRD) device (Empyrean, Malvern, Malvern Panalytical, Malvern, UK) with CuKα radiation (45 kV, 40 mA). The XRD patterns were collected in the 2θ range of 10–70° at a scanning rate of 5°/min, with 10 wt.% zinc oxide incorporated as an internal standard for the semi-quantitative analysis.

Molecular structural characterization was performed using a Fourier-transform infrared spectrometer (FTIR, Nicolet iN10, Thermo Fisher Scientific, Waltham, MA, USA) in the spectral range of 400–4000 cm^−1^, employing potassium bromide as the sample carrier.

The morphological analysis of the surface and cross-sectional features was carried out using a field-emission scanning electron microscope (TESCAN MIRA LMS, TESCAN, Brno, Czech Republic) with a gold sputter coating to ensure adequate conductivity.

## 3. Result and Discussion

### 3.1. Impact of Compaction Degree on the Carbonation Process of Steel Slag Compact

#### 3.1.1. Temperature Variation in Carbonation Environment for Steel Slag Compacts with Different Compaction Degrees

**Figure 3:**
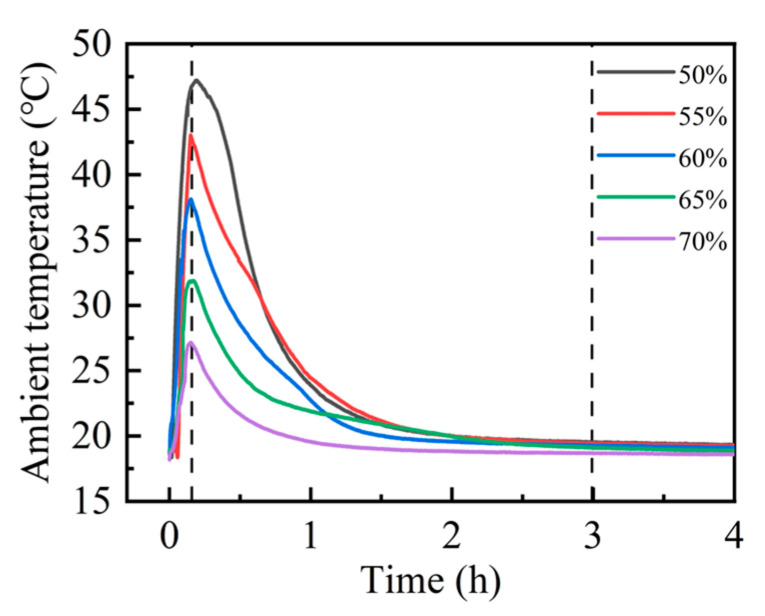
Compact carbonation ambient temperature curves at different compaction degrees.

Upon exposure to carbon dioxide, steel slag compacts with different compaction degrees exhibited rapid exothermic carbonation reactions, resulting in significant thermal release and the consequent elevation in environmental temperature [30,31]. The thermal evolution during the carbonation process was continuously monitored using a precision temperature logger, enabling the real-time tracking of temperature fluctuations. As illustrated in Figure 3, the carbonation environment temperature demonstrated a characteristic thermal profile, reaching its maximum value approximately 15 min after reaction initiation. Subsequently, the temperature gradually decreased, stabilizing at ambient conditions after approximately 3 h, indicating the establishment of thermal equilibrium. Notably, the peak reaction temperature achieved at 15 min exhibited compaction degree dependency. The experimental data revealed an inverse correlation between the compaction degree and peak carbonation temperature. Specifically, the 50% compaction specimen attained the highest peak carbonation temperature of 47.0 °C, while the 70% compaction specimen exhibited the minimum peak temperature of 27.0 °C. This thermal behavior suggests that the increased compaction degree corresponds to reduced carbonation reaction intensity, as evidenced by the progressive decrease in peak temperatures with higher compaction levels.

#### 3.1.2. Synergistic Effect of Compacting Degree and Carbonation Duration on the Performance of Steel Slag Compact

**Figure 4:**
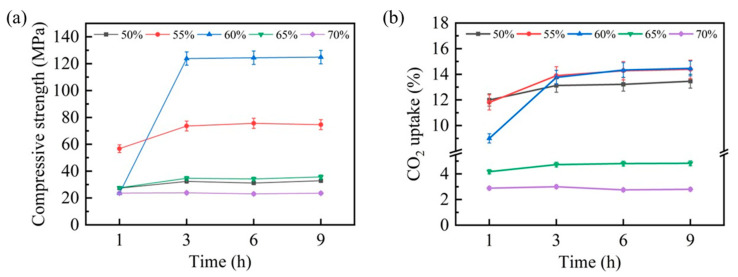
Compressive strength (**a**) and CO_2_ uptake (**b**) of carbonated steel slag compacts at various carbonation times and compaction degrees.

The carbonation behavior of steel slag compacts exhibited significant compaction degree dependency, profoundly affecting their mechanical properties and CO_2_ uptake capacity. To systematically evaluate these effects, comprehensive assessments of the compressive strength and CO_2_ uptake capacity were conducted, with the results presented in Figure 4. Figure 4a illustrates the temporal evolution in the compressive strength for steel slag compacts with varying compaction degrees. Notably, compacts with 50%, 65%, and 70% compaction degrees demonstrated minimal strength enhancement within the initial hour of carbonation, whereas compacts with 55% and 60% compaction degrees exhibited substantial strength improvements. This mechanical enhancement can be attributed to the pore-filling effect of carbonation products formed through the steel slag–CO_2_ reaction, which increases matrix densification and consequently improves compressive strength. After 3 h of carbonation, the compressive strength of steel slag compacts with different compaction degrees stabilized, correlating well with the thermal equilibrium state observed previously.

The CO_2_ uptake capacity, presented in Figure 4b, revealed an inverse relationship between the compaction degree and carbonation efficiency. During the initial carbonation stage (1 h), the steel slag compacts with lower compaction degrees (50% and 55%) exhibited a superior CO_2_ uptake capacity. This capacity progressively diminished with an increasing compaction degree, particularly at 65% and 70% compaction levels, suggesting that excessive compaction impedes carbonation reaction kinetics. Interestingly, after 3 h of carbonation, the CO_2_ uptake capacities of steel slag compacts with 50%, 55%, and 60% compaction degrees converged, while their compressive strength performance remained distinct. This divergence highlights the significant influence of the compaction degree on the mechanical performance of carbonated steel slag compacts.

### 3.2. Impact of Compacting Degree on Carbonation Performance of Steel Slag Compact

The preceding analysis unequivocally demonstrates the significant influence of the compaction degree on the carbonation kinetics and resultant properties of steel slag compacts. Furthermore, distinct performance evolution patterns were observed across different compaction levels, warranting a detailed investigation into the mechanical properties of both pre- and post-carbonation compacts.

**Figure 5:**
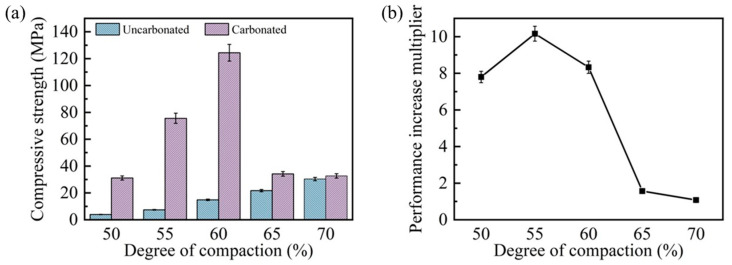
Compressive strength (**a**) and strengthening degree (**b**) of steel slag compacts with different compactness before and after carbonation.

Figure 5 presents a comprehensive comparison of the compressive strength and the corresponding enhancement ratios for steel slag compacts with different compaction degrees before and after 9 h of carbonation. As illustrated in Figure 5a, the pre-carbonation compressive strength exhibited a positive correlation with the compaction degree, increasing from 4.0 MPa at 50% compaction to 30.3 MPa at 70% compaction, representing a 6.6-fold enhancement. However, the compressive strength of post-carbonation steel slag compacts exhibited an inverse relationship, peaking at 124.4 MPa for 60% compaction before declining with further increases in the compaction degree. Notably, while the 50% compaction specimen achieved a 6.8-fold strength increase (31.15 MPa) after carbonation, its strength remained inferior to compacts with intermediate compaction degrees (55–60%) due to inherent porosity limitations. The steel slag compacts with 70% compaction demonstrated a minimal strength enhancement (7.81%, to 32.7 MPa), indicating compromised carbonation efficiency at higher compaction degrees.

**Figure 6:**
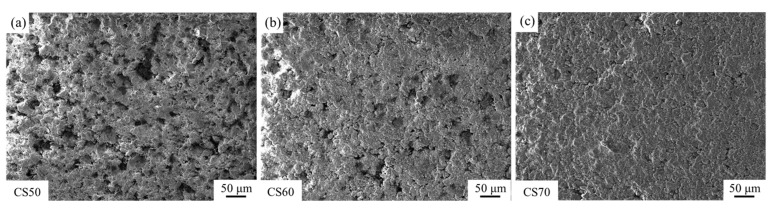
Surface SEM of steel slag compacts with different compactness after carbonation, (**a**) CS50, (**b**) CS60, (**c**) CS65.

Figure 5b shows that the enhancement ratios of compressive strength after carbonation mirrored the trend of the compressive strength values after carbonation. This behavior can be attributed to the dual effects of the compaction degree on the matrix degree and gas permeability. While increased compaction reduces porosity and enhances structural integrity, excessive compaction impedes CO_2_ diffusion, thereby inhibiting the carbonation progression. This phenomenon is visually corroborated in Figure 6, which displays the formation of a thick carbonation layer on the compact surfaces, effectively restricting gas transport pathways. These findings suggest that optimal carbonation performance requires a balance between the matrix density and gas permeability. Intermediate compaction degrees (e.g., 60%) facilitate both adequate structural integrity and sufficient CO_2_ diffusion, resulting in maximum strength enhancement. Conversely, extreme compaction levels (either too low or too high) adversely affect carbonation efficiency and mechanical performance, demonstrating the importance of compaction degree optimization in steel slag carbonation processes.

### 3.3. Mechanism of Influence of Compaction Degree on Carbonation Properties of Steel Slag Compact

**Figure 7:**
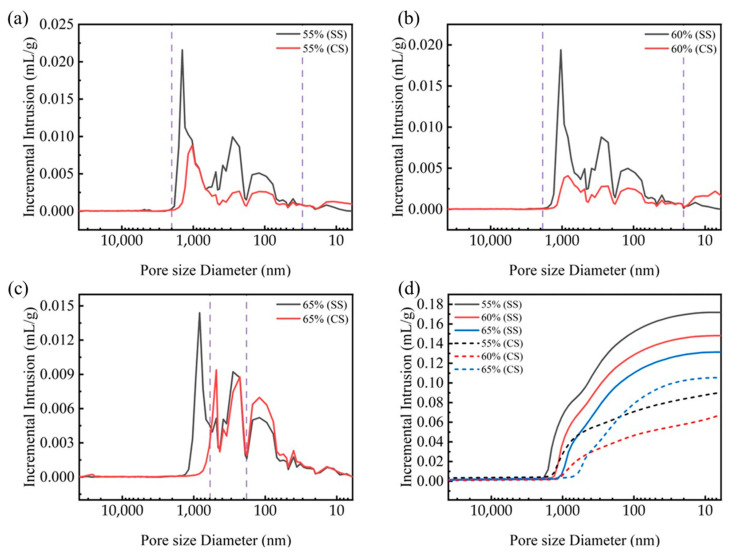
Pore distribution and cumulative intrusion (**d**) of steel slag compacts 6 h after curing at varying compaction degrees, (**a**) CS55, (**b**) CS60, (**c**) CS65.

To elucidate the influence of the compaction degree on the microstructural evolution during carbonation, the pore structure was analyzed using mercury intrusion porosimetry (MIP). MIP tests were conducted on steel slag compacts with compaction degrees of 55%, 60%, and 65% before (SS) and after carbonation (CS). The results, shown in Figure 7, reveal distinct pore size distribution patterns across different compaction levels. For steel slag compacts with 55% and 60% compaction degrees, the pore size distributions exhibit similar transition trends following carbonation. A substantial reduction in pore volume was observed within the pore size range of 20 nm to 2000 nm, accompanied by a moderate increase in the 5–20 nm range. This microstructural evolution can be attributed to the pore-filling mechanism of carbonation products, which effectively transform larger pores to smaller ones though precipitation reactions [32,33,34].

In contrast, the 65% compaction specimen exhibits unique pore structure characteristics. While a decrease in pore volume was noted in the 500–1200 nm range, a substantial increase occurred in the 20–500 nm range. This phenomenon suggests that the excessive compaction restricts the carbonation reaction primarily to the surface region, where the carbonation products accumulate and occlude surface pores. Consequently, this surface densification inhibits the inward diffusion of carbon dioxide into the interior matrix, suppressing the carbonation reaction within the bulk material.

Figure 7d illustrates the evolution of the total cumulative intrusion volume for steel slag compacts with different compaction degrees with a comparison between pre- and post-carbonation states. It can be seen that before carbonation, the pore volume of the steel slag compact gradually decreases as the compaction level increases, indicating that increased compaction effectively compresses the interparticle voids and enhances matrix densification. The post-carbonation analysis revealed a non-monotonic relationship between the compaction degree and pore volume reduction. Below the critical compaction threshold (65%), increased compaction promotes the pore filling by carbonation products, resulting in enhanced compressive strength. However, beyond this threshold, excessive compaction restricts gas diffusion, reducing the carbonation efficiency and compromising the pore optimization effect. This phenomenon is quantitively supported by the mercury intrusion data presented in Table 3. Optimal compaction conditions enable effective pore filling by carbonate products, leading to simultaneous improvements in multiple microstructural parameters, including reduced porosity, increased bulk density, and decreased average pore size. These microstructural modifications collectively contribute to the enhanced mechanical performance observed at intermediate compaction degrees.

**Figure 8:**
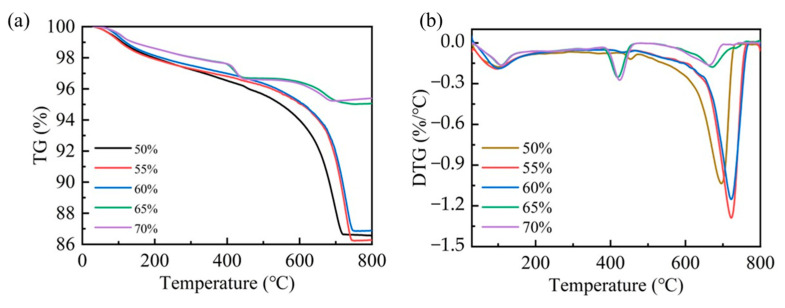
TG (**a**) and DTG (**b**) patterns for steel slag compacts of varying compactness.

The carbonation efficiency of steel slag compacts exhibits compaction degree dependency. To further elucidate the carbonation mechanism, a thermogravimetric analysis (TGA) was employed to quantify the carbonation products of steel slag compacts with different compaction degrees. As shown in Figure 8, the predominant mass occurred within the temperature range of 300–800 °C, corresponding to the thermal decomposition of carbonates. This characteristic mass loss serves as a reliable indicator for quantifying carbonation product formation [23,35]. The quantitative analysis of the mass loss data presented in Table 4 revealed that the amount of carbonation products decreases as compaction increases. Specifically, the steel slag compacts with intermediate compaction levels of 50%, 55%, and 60% demonstrated a significantly higher carbonate content compared to those with higher compaction levels of 65% and 70%. This observation indicates that optimal compaction facilitates more complete carbonation reactions, resulting in enhanced carbonation formation and consequent improvement in mechanical properties. Conversely, excessive compaction suppresses the carbonation process through restricted CO_2_ diffusion, thereby diminishing both the reaction efficiency and mechanical performance enhancement.

**Figure 9:**
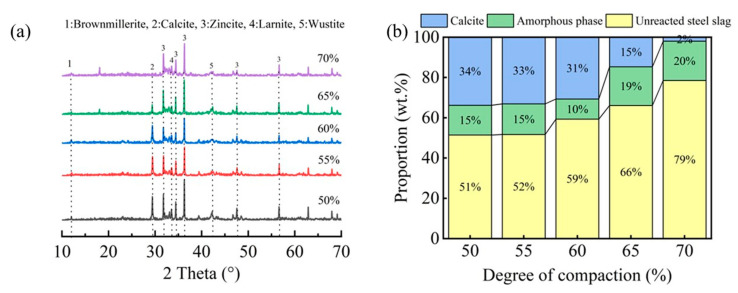
XRD patterns (**a**) and the ratio of mineral phases (**b**) in steel slag compacts at varying degrees of compactness.

Figure 9 displays the XRD patterns and corresponding mineral phase composition of carbonated steel slag compacts with different compaction densities. As illustrated in Figure 9a, the characteristic calcite peak intensity, indicative of the carbonation product formation, exhibits a progressive decrease with an increasing compaction degree. Notably, compacts with lower compaction degrees (50–60%) demonstrated significantly higher calcite peak intensities compared to their highly compacted counterparts (65–70%).

**Figure 10:**
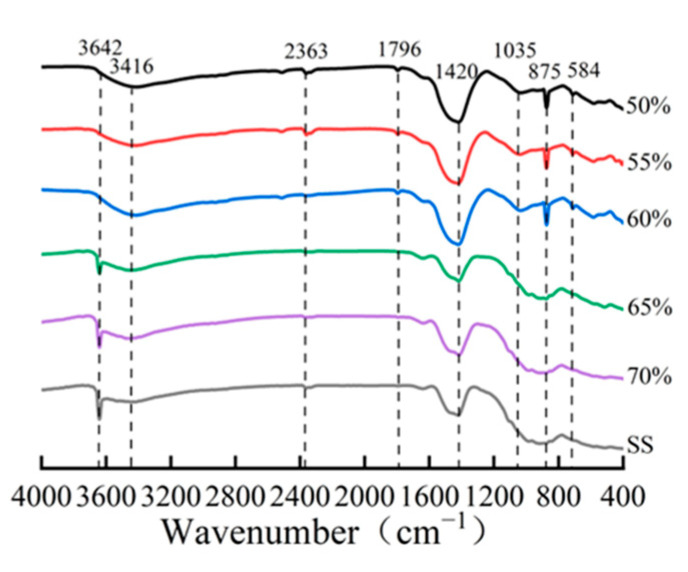
IR patterns of compacted carbonated steel slag at varying degrees of compactness.

The quantitative phase analysis in Figure 9b reveals a critical compaction threshold at approximately 65%. Below this threshold, the compacts exhibit a higher carbonation degree and correspondingly greater carbonate product formation. However, beyond this critical value, both the carbonation degree and calcite content showed a marked reduction. This observation is further corroborated by FTIR analysis (Figure 10), where the characteristic calcium carbonate absorption band at 875 cm^−1^ [17,25] showed significant attenuation at compaction degrees exceeding 65%. These complementary analytical results consistently demonstrate that excessive compaction inhibits carbonation product formation through restricted CO_2_ diffusion and limited reaction space.

**Figure 11:**
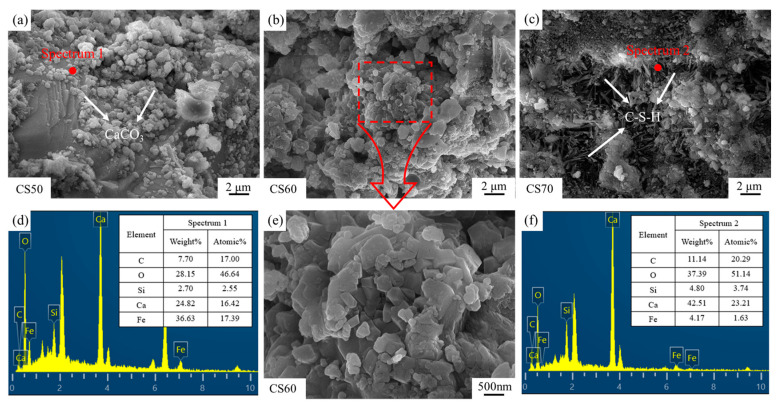
SEM images and EDS spot scan findings for carbonated steel slag compacts of varying densities, (**a**) CS50, (**b**) CS60, (**c**) CS70, (**d**) Spectrum 1, (**e**) Enlarged CS60, (**f**) Spectrum 2.

Figure 11 illustrates the microscopic morphologies of carbonated and cured steel slag compacts with different compaction degrees. SEM micrographs in Figure 11d,f clearly identify the predominant carbonation product as calcium carbonate and the main hydration product as calcium silicate hydrate (C-S-H) [36,37,38]. As evidenced in Figure 11a, low compaction degrees lead to discrete and sparsely distributed calcium carbonate particles. This discontinuous morphology fails to establish an effective filling network, thereby limiting the reinforcing potential of the carbonation products. Consequently, the mechanical property improvement remains suboptimal at low compaction degrees due to insufficient matrix densification and poor interfacial bonding between reaction products and the slag matrix.

As the compaction degree increases, a progressive reduction in pore volume (the pore volume refers to the total void space within a unit mass or unit volume of porous material, typically expressed in units of cm^3^/g or mL/g, serving as a critical parameter for characterizing the porous structure of materials) is observed in the steel slag compacts. SEM micrographs in Figure 11b,e indicate the formation of interconnected calcium carbonate networks, where particle aggregation and interlocking significantly enhance mechanical strength. However, at elevated compaction degrees (Figure 11c), the formation of a dense surface layer impedes the inward diffusion of carbon dioxide, resulting in incomplete internal carbonation and reduced calcium carbonate precipitation. Under these conditions, the microstructure becomes predominantly characterized by needle-like hydration products (C-S-H), which exhibit limited strengthening effects [39,40].

**Figure 12:**
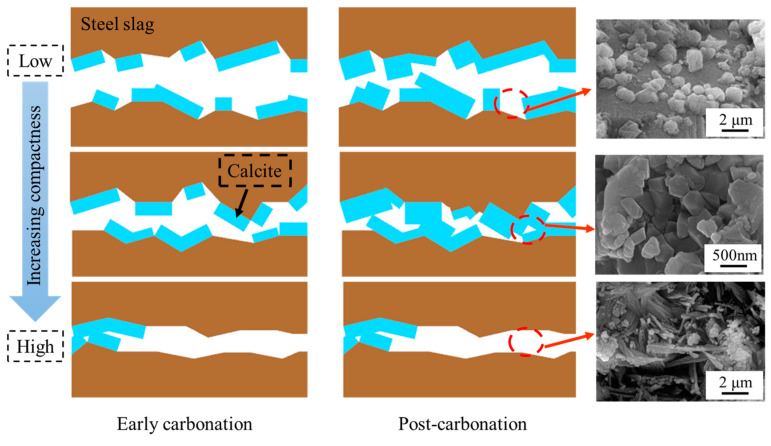
Mechanism diagram of influence of compaction degree on carbonation properties of steel slag compact.

The mechanistic influence of the compaction degree on the carbonation performance of steel slag compacts is schematically illustrated in Figure 12. In the early stages of carbonation, the reaction between steel slag and carbon dioxide generates substantial amounts of calcium carbonate, which effectively fills the porous matrix [41]. For compacts with low to medium compaction degrees, this pore-filling mechanism enhances the matrix density, leading to significant improvement in mechanical properties. Nevertheless, in highly compacted steel slags, although calcium carbonate initially fills available pores, it simultaneously occludes the diffusion pathways of carbon dioxide, thereby inhibiting subsequent carbonation reactions and limiting overall carbonation efficiency.

With a prolonged carbonation duration, low-compaction steel slag compacts continue to accumulate carbonation products. Nevertheless, the inherent large interparticle voids result in the formations of discrete and isolated calcium carbonate particles, preventing the development of a continuous reinforcing network. This morphological limitation restricts the enhancement of mechanical properties. At optimal compaction degrees, the pores with reduced volume fractions facilitate the formation of interconnected calcium carbonate networks through particle accumulation and bonding, resulting in a substantial structure densification and significant improvement in mechanical properties.

For steel slag compacts with excessive compaction degrees, the formation of carbonation products at the initial stage prematurely seals the diffusion channels of carbon dioxide, restricting the carbonation reactions of the interior matrix, and consequently limiting the mechanical performance enhancement. The mechanistic analysis reveals that the compaction degree governs the carbonation efficiency and product distribution through pore structure modulation, ultimately determining the mechanical performance of carbonated steel slag compacts. An optimal pore structure achieves a balance between accommodating sufficient calcium carbonate precipitation and maintaining adequate CO_2_ permeability, enabling deeper carbonation reactions and optimal performance enhancement.

## 4. Conclusions

(1) As the compaction degree increases, the peak temperature within the carbonation environment gradually declines, accompanied by a reduction in the intensity of the early-stage carbonation reaction. Post-carbonation, the compressive strength exhibits an initial increase followed by a decrease with an increasing compaction degree, reaching an optimal compressive strength of 124.41 MPa at a compaction degree of 60%.
(2) The pore size distribution of carbonated steel slag compacts demonstrates significant variation with compaction degrees. At low to medium compaction degrees, the pore size of steel slag compacts decreases markedly, leading to a substantial improvement in pore structure. However, at excessively high compaction degrees, the calcium carbonate formed on the surface acts as a barrier impeding gas diffusion. This obstruction suppresses the carbonation process and ultimately diminishes the effectiveness of pore refinement.
(3) At lower compaction degrees, the pores of steel slag compacts are larger, and the filling effect of carbonation products is limited. On the other hand, at excessively high compaction degrees, the calcium carbonate particles generated during the early stages of carbonation can block the gas diffusion channels. An optimal compaction degree allows for sufficient filling by calcium carbonate products while preventing premature channel blockage, thereby achieving optimal performance enhancement.
(4) Meanwhile, the carbonation products of steel slag compacts at lower compaction levels disperse in an isolated manner. When the compaction degree is increased to an appropriate level, the calcium carbonate particles accumulate and bond together, substantially enhancing the performance of the steel slag compacts.

The findings of this study demonstrate that the compaction degree significantly influences the performance of steel slag compacts post-carbonation. Different compaction degrees can lead to variations in the internal structure of steel slag, thereby affecting its physical and chemical properties. For instance, a higher compaction degree can reduce the porosity of steel slag, thereby increasing its density and strength. However, excessively high compaction can lead to excessive internal stress within the material, which may, in turn, decrease its durability and stability. Therefore, in large-scale production, the precise control of the compaction degree can effectively reduce energy consumption and time wastage, lower production costs, and improve economic efficiency. Simultaneously, optimizing compaction can enhance CO_2_ absorption efficiency, enabling companies to reduce their carbon footprint, and accelerate their transition towards sustainable practices. This provides potential application value for the future utilization of steel slag, a solid waste material, in the construction industry.

## Figures and Tables

**Table 1:** Chemical composition of steel slag (wt.%).

CaO	MgO	Al_2_O_3_	Fe_2_O_3_	MnO	SiO_2_	P_2_O_5_	TiO_2_	Loss of Ignition
40.55	6.69	8.20	22.69	4.48	13.17	1.44	1.12	1.66

**Table 2:** Powder mass required at different compaction degrees.

Degree of Compaction (%)	Steel Slag Powder (g)	W/s (1:10) Mixed Powder (g)
50	11.15	12.27
55	12.26	13.49
60	13.38	14.72
65	14.49	15.94
70	15.61	17.17

**Table 3:** Examination of pore architecture in steel slag compacts 6 h after curing.

Compact Number	Porosity (%)	Bulk Density (g/mL)	Apparent Density (g/mL)	Average Pore Diameter (nm)
SS55	36.10	2.10	3.29	199.51
SS60	32.48	2.19	3.25	175.81
SS65	30.24	2.30	3.30	145.12
CS55	21.07	2.32	2.94	78.29
CS60	16.79	2.46	2.92	59.44
CS65	25.64	2.43	3.27	104.15

**Table 4:** Mass loss of steel slag compacts with different compacted degrees between 300 °C and 800 °C.

Degree of Compaction (%)	Mass Loss Between 300 °C and 800 °C (%)
50	10.92
55	11.43
60	11.32
65	2.29
70	1.95

## Data Availability

The original contributions presented in this study are included in the article. Further inquiries can be directed to the corresponding author.
